# The prevalence of risk for hearing impairment in newborns with congenital syphilis in a newborn hearing screening program (NHS)

**DOI:** 10.3389/fpubh.2023.1214762

**Published:** 2023-09-21

**Authors:** Thalita da Silva Oliveira, Monique Ramos Paschoal Dutra, Aryelly Dayane da Silva Nunes-Araujo, Aline Roberta Xavier da Silva, Gabriel Barros Lins Lelis de Oliveira, Gleyson José Pinheiro Caldeira Silva, Ricardo Alexsander de Medeiros Valentim, Sheila Andreoli Balen

**Affiliations:** ^1^Laboratory of Technological Innovation in Health - LAIS, Federal University of Rio Grande do Norte, Natal, Rio Grande do Norte, Brazil; ^2^Januario Cicco Maternity School, Federal University of Rio Grande do Norte, Natal, Rio Grande do Norte, Brazil

**Keywords:** hearing, newborns, screening, hearing loss, congenital syphilis

## Abstract

**Objective:**

To study the prevalence of risk for hearing impairment in neonates with congenital syphilis in a newborn hearing screening program.

**Study design:**

The study design is retrospective, documentary, and is cross-sectional. The sample consisted of newborns who were born between January 2019 and December 2021 and who underwent neonatal hearing screening in a public maternity hospital. Demographic data and the presence and specification of risk indicators for hearing impairment (RIHL) were collected. In retest cases, the results and the final score were also collected. For data analysis, the Kruskal–Wallis and Conover-Iman *post-hoc* tests were used, comparing the groups that passed and failed the hearing screening that had RIHL, using a significance level of *p* of <0.5.

**Results:**

Among the RIHL observed in the sample, prematurity was more frequent in newborns who passed the screening (55.26%) than in those who failed the test (45.67%). Congenital syphilis was the ninth most frequent RIHL (8.04%) among the newborns who passed the test and the 15th factor (3.03%), with the highest occurrence in those who failed the hearing screening. When comparing the two groups (pass and fail), we found significant differences (*p * < 0.05) between them.

**Conclusion:**

Congenital syphilis was the ninth risk indicator for the most common hearing impairment and, in isolation, did not present a risk for failure in neonatal hearing screening. Notably, congenital syphilis can cause late hearing loss during child development. Thus, there is an indication of audiological monitoring of these neonates.

## Introduction

Congenital syphilis is an infection caused by *Treponema Pallidum* that can be transmitted via the placenta or at the time of delivery when the infection in the pregnant woman is not properly treated at any stage of pregnancy. As it is often asymptomatic, the detection of congenital syphilis in children depends on tests performed in the maternity ward. This detection is crucial because, when not properly treated, it can cause metabolic alterations, neurological alterations, prematurity, low birth weight or low weight during the child's first years of life (1).

Worldwide, syphilis is estimated to occur in approximately one million pregnancies each year, resulting in more than 350,000 adverse pregnancy outcomes (1). Regarding congenital syphilis, we can highlight Brazil as one of the countries with the most cases in the world, with approximately 896 cases per 100,000 live births (2). The increase in cases of syphilis in pregnancy during the last few years has been a cause for concern in the Brazilian public health sector due to the potential for infection in the fetus (3).

Congenital syphilis is one of the congenital infections considered to be a risk indicator for hearing impairment (4), and the identification of hearing loss (whether profound, moderate, mild, or unilateral) can occur through newborn hearing screening programs (4, 5). In the case of syphilis, alterations can be detected during hearing screening or auditory monitoring when periodic hearing procedures are carried out in the form of a cross-check with electrophysiological, electroacoustic, and behavioral evaluations aimed at monitoring the child's development relative to hearing (6). Current studies indicate that there is no difference in the responses to electrophysiological procedures during the first months of life between children without risk indicators and those with congenital syphilis. However, there is a need to examine these children during the first 2 years of life because there is a higher risk of hearing loss in the future (7).

In addition to syphilis, newborns with the presence of other risk indicators, such as prematurity, hyperbilirubinemia, congenital or viral infections, use of ototoxic drugs, and staying in the Neonatal Intensive Care Unit (NICU) for >5 days, among others, must be monitored after hearing screening, regardless of failure or not in the procedure (4, 8). Moreover, continuously evaluating the quality indicators of neonatal hearing screening programs proposed by international guidelines (4) is of great importance for us to monitor how the coverage of hearing screening is found and the speed with which newborns are referred both to hearing monitoring and diagnosis.

Therefore, identifying risk indicators for hearing impairment is paramount for children's hearing health and consequently strengthens the promotion, prevention, and follow-up activities in the Comprehensive Care Network for children in the health system. Thus, the objective of the study was to study the prevalence of risk for hearing impairment in neonates with congenital syphilis in a newborn hearing screening program (NHSP).

## Methods

The present study has a retrospective, documental, cross-sectional study design that was carried out at a public maternity hospital's Newborn Hearing Screening Sector. This study was approved by the Committee of Ethics of the University Hospital Onofre Lopes, Federal University of Rio Grande do Norte, Natal, Rio Grande do Norte, Brazil, number 4,648,404. This maternity hospital is a reference in maternal and child health in the state because it has Neonatal and Maternal Intensive Care Unit beds and is responsible for the birth of approximately 3,600 newborns a year (9).

The sample comprised neonates who were born in the maternity hospital between January 2019 and December 2021 and who had neonatal hearing screening. Those born in other institutions were excluded from the survey, making the size of the final sample 7,879 records. This sample corresponded to 66.69% of live births during the study period at this maternity hospital.

NHS was performed during the mother's and neonate's hospitalization or at the institution's outpatient clinic by one of the two audiologists in the program. The equipment used was the Madsen^®^ AccuScreen, which was duly calibrated, and the exam was the Transient Stimulus Evoked Otoacoustic Emissions (TEOAE).

The probe test of the equipment was performed every day before the test was applied to the infants. In this way, it was inspected and connected. Afterward, the infants' data were entered into the equipment's software.

The infants were comfortably positioned on their mother's lap in natural sleep so that their ears were accessible for inserting the probe into the external auditory meatus. The audiologist had performed a prior inspection of the acoustic meatus to identify the presence of cerumen or other residues at the entrance of the meatus. Subsequently, the audiologist would insert the auricular tip into the appropriately sized probe for each external acoustic meatus so that it would be in a proper position. During this insertion procedure, the audiologist carefully pulled the external acoustic meatus backward and slightly downward with slight pressure. Afterward, the audiologist visually checked whether the fit was correct using a horizontal bar that highlighted this functionality. When starting the TEOAE test, this equipment first performs the probe calibration, and as soon as it completes the calibration, the test starts automatically in each ear, with both performed one after the other. In this equipment, the pass response is determined by a statistical algorithm based on the weighted average, which ensures detection with proven high specificity and sensitivity. The “pass” result in the screening according to the parameters in the equipment manual presents the following parameters: eight peak frequency responses, an artifact rate of <20%, and probe stability >80%. If one of these parameters is not reached, it is considered a “failure” in the screening.

**Figure 1:**
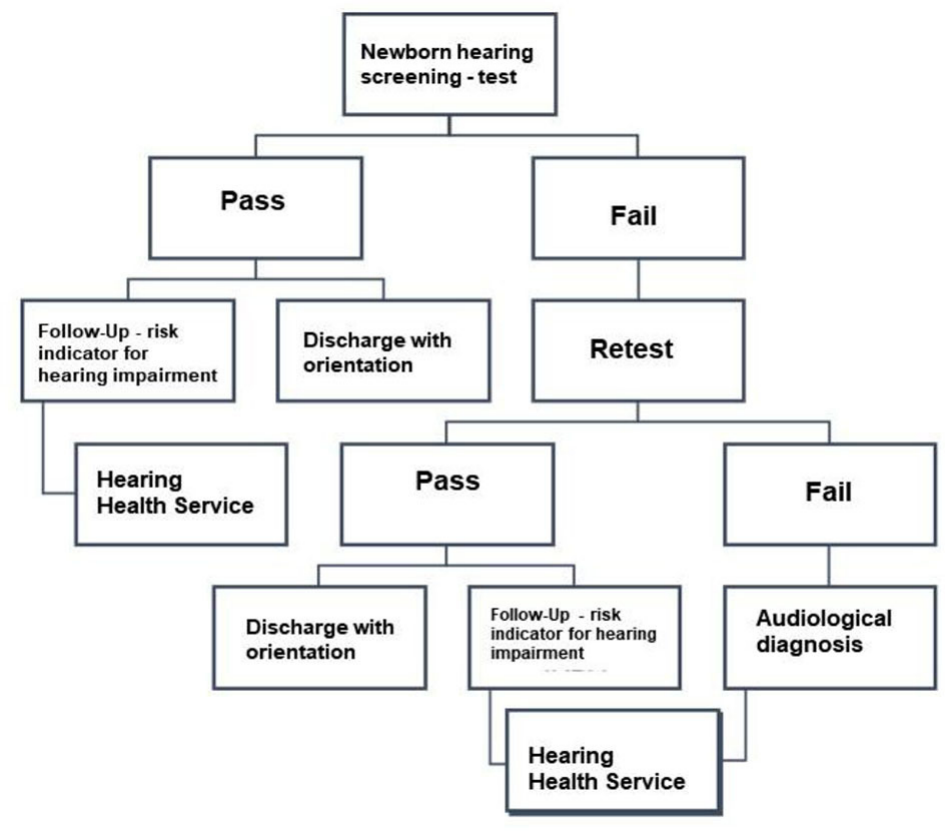
A flowchart of the maternity newborn hearing screening program. RIHL, Risk Indicator for Hearing Loss; SSA, Hearing Health Service; CER, Center Specialized in Rehabilitation.

The NHS was carried out in two stages. In the first test call, it was considered a “pass” when the newborn obtained the presence of otoacoustic emissions in both ears and “fail” when the emissions were not present in at least one ear. When “failure” occurred, a retest was scheduled at the maternity clinic after ~15–30 days. If there was a new failure in the retest, the baby was referred for an audiological diagnosis at the Hearing Health Service (SSA) or Center Specialized in Rehabilitation (CER) to perform an audiological diagnosis. If the newborn had a risk indicator for hearing impairment (RIHL), they were also referred to the reference service for hearing monitoring, which consists of audiological evaluation and monitoring of hearing and language development. Finally, when the newborn had no risk indicator and the result was “pass,” the child was discharged with guidance, and the caregiver received verbal guidance on the child's auditory and language development. The flowchart is shown in Figure 1.

Data collection from medical records occurred from October 2021 to February 2022 through the consultation of the care records and the sector's database.

Owing to the absence of a computerized database, the NHS sector organizes the data necessary for evaluating the neonatal hearing screening program in an Excel spreadsheet. The data extracted were the date of screening, birth date of the neonate, neonate's gender, municipality where the mother lives, presence and specification of RIHL, screening result (passed bilaterally, failed right ear, or failed left ear), and management (discharge with counseling, retesting, hearing monitoring, or hearing diagnosis). In cases of retesting, data on attendance (yes or no), the result (bilateral pass, right ear failure, or left ear failure), and the final management (discharge with counseling, hearing monitoring, or hearing diagnosis) were also collected.

A survey of the patient's clinical history was carried out, considering all risk indicators for hearing impairment as identified in the program and recorded by the medical team responsible for caring for the parturient woman and the neonate. These indicators include altered APGAR scores, arbovirus infections during pregnancy, craniofacial anomalies involving the ear and the temporal bone, severe perinatal anoxia, heredity factors, hyperbilirubinemia, congenital infections (toxoplasmosis, rubella, cytomegalovirus, herpes, syphilis, and HIV), postnatal bacterial or viral infections (cytomegalovirus, herpes, measles, chickenpox, and meningitis), being small for gestational age, weight <1,500 g, prematurity, parental concerns, syndromes often associated with hearing impairment, use of ototoxic drugs, use of mechanical ventilation, and staying in the Neonatal Intensive Care Unit (NICU) for more than 5 days.

All risk indicators were collected from each newborn's medical records at the maternity hospital. Therefore, the identification and diagnosis criteria for each of the criteria followed the good medical practices of this maternity hospital in line with the guidelines recommended by the Ministry of Health of Brazil. With regard to congenital syphilis, the parameters adopted for its diagnosis and treatment followed the Clinical Protocol and Therapeutic Guidelines of the Ministry of Health (10).

It should be noted that, until the year 2019, maternity followed the Brazilian Ministry of Health's Guidelines for Newborn Hearing Screening (2012) (11), similar to the International Joint Committee on Infant Hearing (2007) (12). Following the update of the international recommendations of the same committee in 2019 (4), also followed by a technical note from the Multiprofessional Committee on Hearing Health (2020) (8), the maternity ward currently follows current risk indicators for hearing impairment. Since the study went through the transition years of the protocol concerning the RIHL of the maternity ward, the protocol followed until the year 2019 was recommended in this study since it has a more comprehensive number of indicators. All the mentioned information was organized in a new Excel spreadsheet so that the statistical analyses could be followed. Descriptive and inferential statistical analysis was performed, considering the categorical characteristics of the variables under study (pass or fail on NHS and RIHL).

In the data analysis, the percentage distribution of categorical variables and measures of central tendency and dispersion of continuous variables were performed. For statistical analysis, the Kruskal–Wallis and Conover-Iman's *post-hoc* statistical tests compared the groups that passed and failed hearing screening that had RIHL, using a significance level of *p* of <0.5.

## Results

In the studied period, 11,927 neonates were born at the maternity hospital, of which 7,879 (66.69%) underwent NHSP. Of the screened neonates, 96 (1.14%) reported congenital syphilis as the only RIHL and 90 (1.07%) reported congenital syphilis associated with other RIHL, totaling 186 neonates with congenital syphilis.

Of these, 183 (98.39%) neonates passed the test and retest, two (1.07%) did not show up for the retest, and the screening was not concluded, and one (0.54%) neonate did not pass the test and retest and was referred to the Specialized Care Service for a complete audiological diagnosis. In addition to congenital syphilis, this neonate was premature, had been in the ICU for >5 days, and used ototoxic medication.

**Figure 2:**
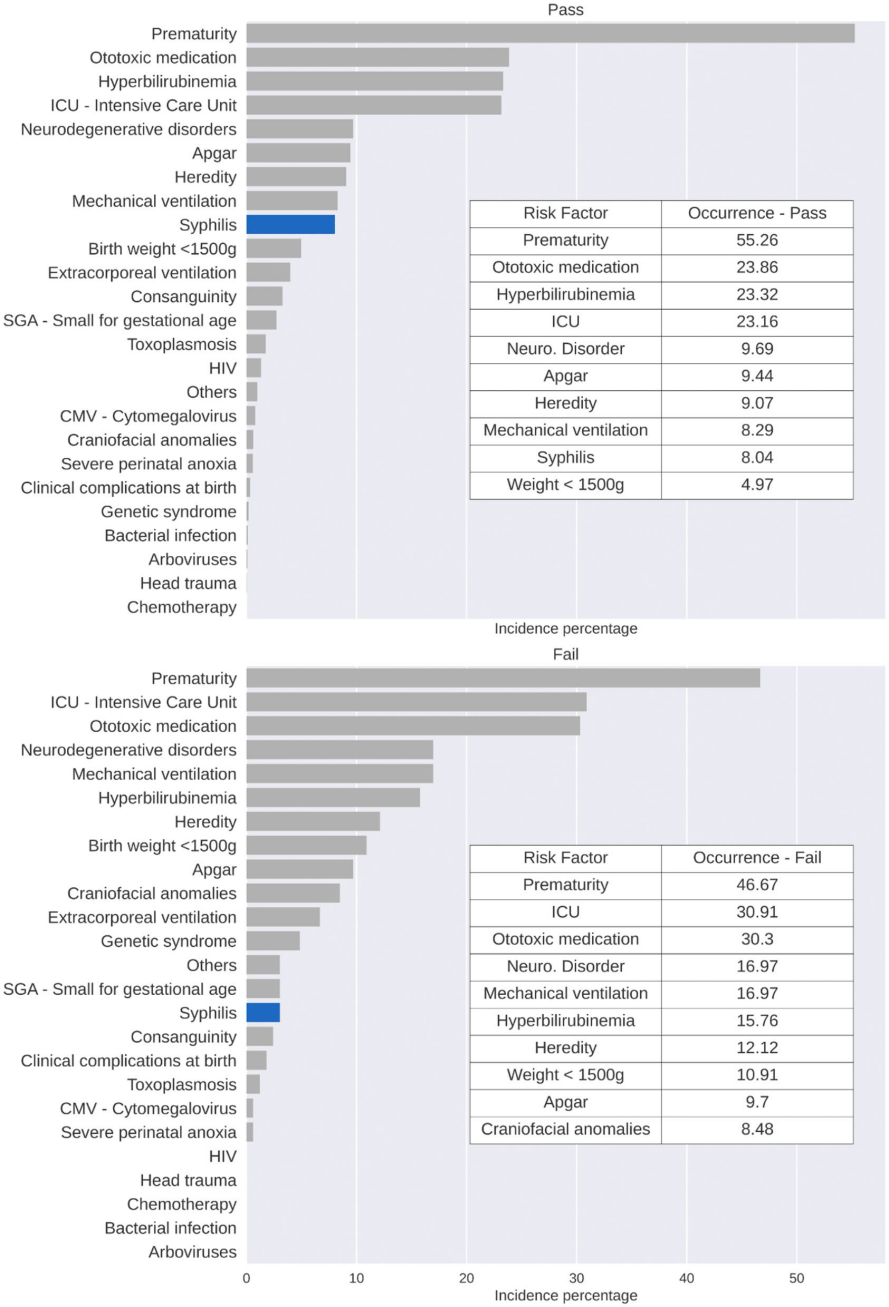
RIHL occurrence percentage by failure-pass, based on the sample of individuals with RIHL, considering the studied period.

The statistical analysis showed that, among the RIHL observed in the sample, prematurity was most prevalent in the group of neonates who were evaluated (55.26%) and those who failed the test (45.67%) (Figure 2); congenital syphilis was the ninth most prevalent RIHL (8.04%) among neonates who passed the test and the 15th most prevalent factor (3.03%) among neonates who failed the hearing screening (Figure 2). When comparing the two groups (pass and fail) using the Kruskal–Wallis test and the Conover-Iman *post hoc* test, we found a significant difference (*p * < 0.05) between them.

**Figure 3:**
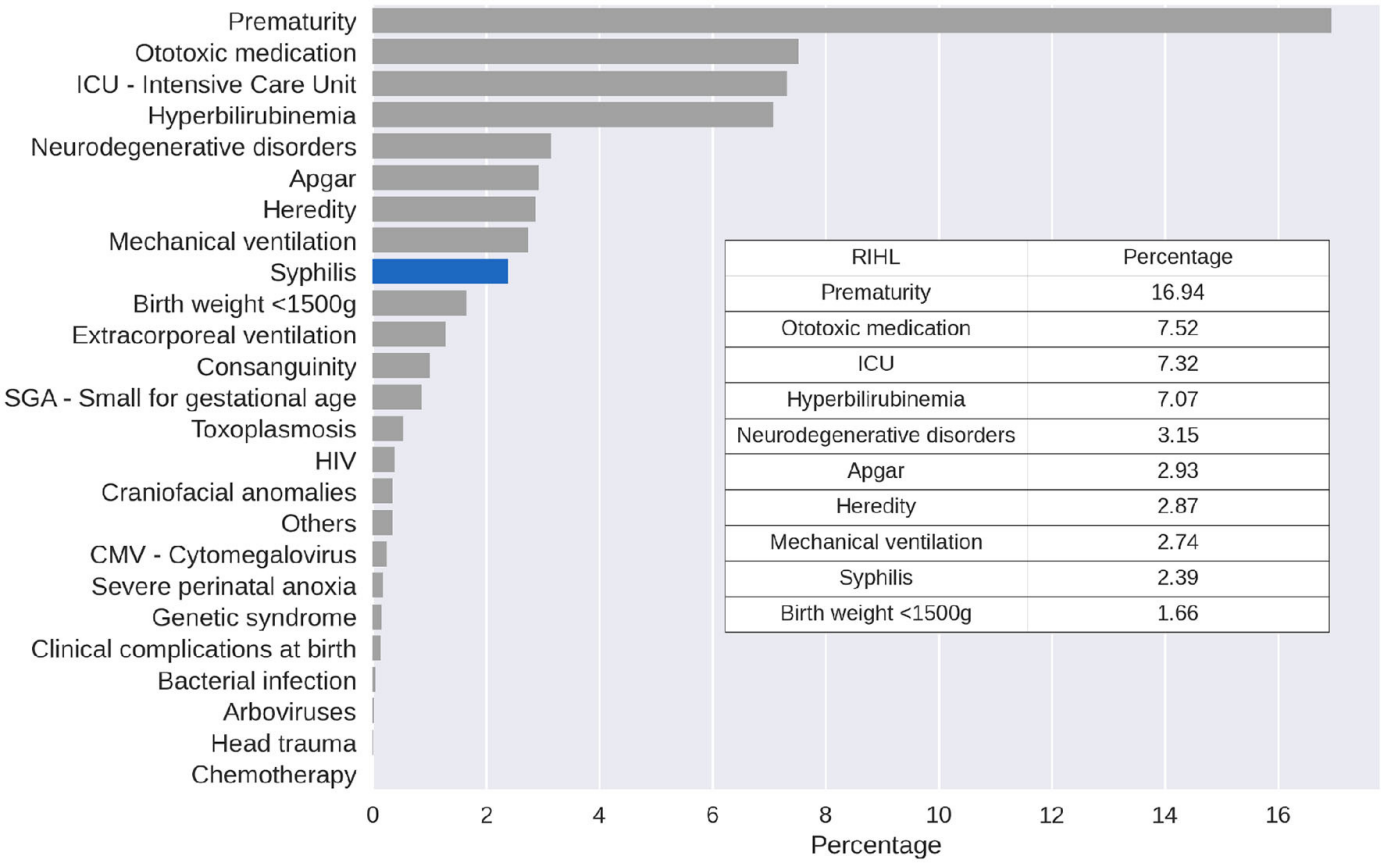
RIHL incidence percentage considering the whole sample for the 3 observed years.

The analysis also showed that the percentage of RIHL incidence throughout the sample during the 3 years observed, when the 10 risk indicators for hearing impairment with the highest incidence were found, with prematurity, ototoxic medication, and an ICU stay of >5 days being the most common (Figure 3).

**Figure 4:**
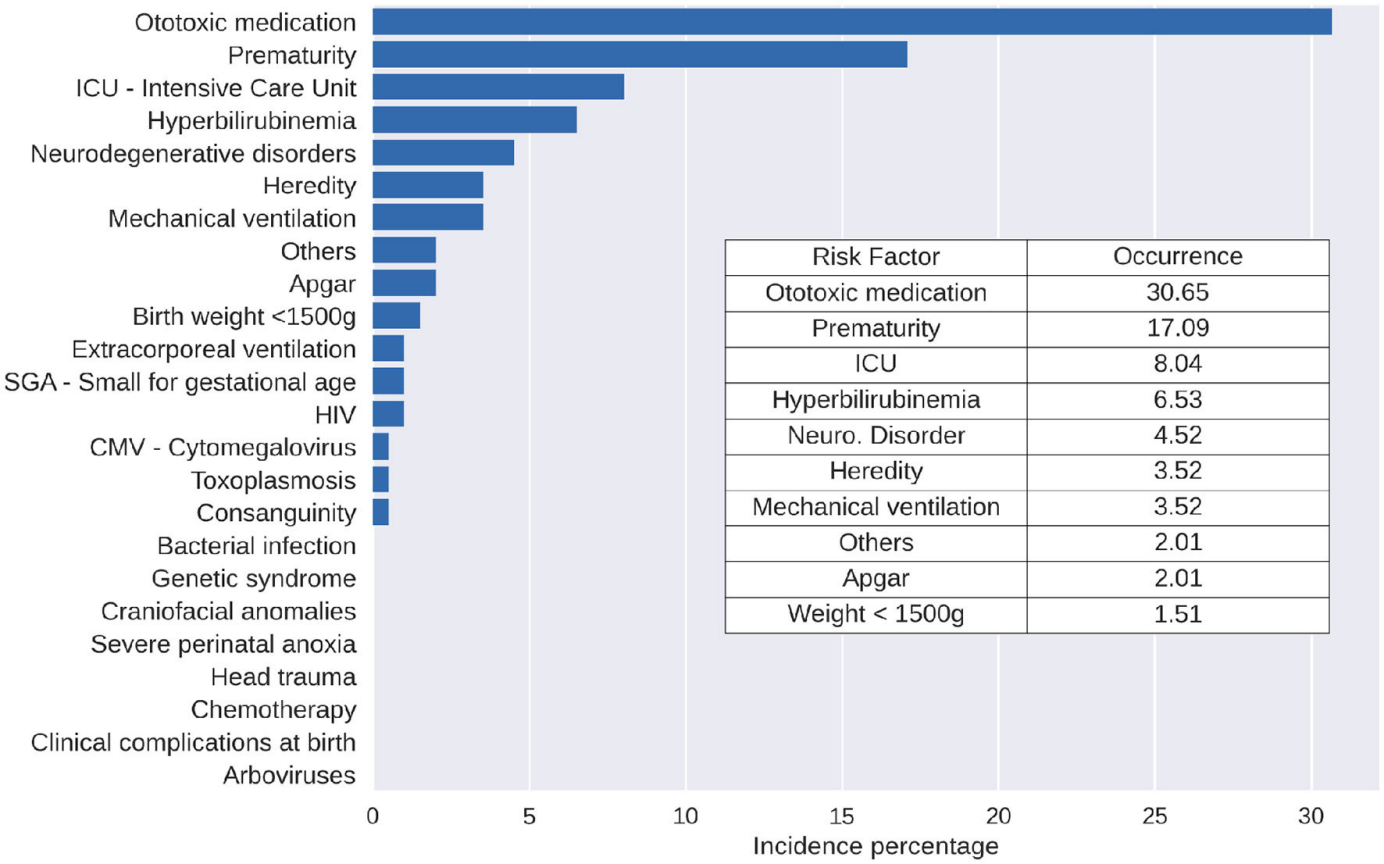
Description of the RIHL occurrence percentage in the presence of syphilis, considering the studied period.

Furthermore, statistical analysis showed that, in the presence of syphilis, the main accompanying risk factor is ototoxicity, followed by prematurity and an ICU stay of >5 days (Figure 4).

## Discussion

Congenital syphilis is a RIHL, according to national and international scientific recommendations. In the sample studied, no neonate with isolated congenital syphilis failed the neonatal hearing screening test. Even so, these infants should be referred for hearing monitoring and medical follow-up since they may develop signs and symptoms later in life, regardless of the first evaluation and/or treatment in the maternity hospital (9, 11).

It was observed that the coverage of neonatal hearing screening performed in the program studied did not reach the rate recommended by the Joint Committee on Infant Hearing (4), staying below 95%. Some factors may be related, such as the SARS-COV pandemic, the family's difficulty in moving around, a lack of financial resources, and illnesses.

Regarding the RIHL, prematurity, hyperbilirubinemia, use of ototoxic medication, and a stay at the NICU for more than 5 days were the most frequent in the sample, both in neonates who passed or failed the screening, which does not differ from what has been found in other recent studies (13–16). These indicators may be related to premature neonates' need for intensive care and medication use.

As for congenital syphilis, it was noticed in the sample studied that congenital infection was the most frequent, but in isolation, it did not present a risk for failure in neonatal hearing screening. In Brazil, over the past 5 years, a steady increase in the number of cases of syphilis in pregnant women, congenital syphilis, and acquired syphilis has been observed (9). This increase is reflected in NHSP programs that present congenital syphilis as one of the most prevalent RIHLs in neonates (17–19). Another factor contributing to congenital syphilis being the most prevalent among congenital infections may be due to the public policy in Brazil that, since 2005 (20), has made notification of syphilis compulsory in pregnant women.

Congenital neurosyphilis is an infection of the central nervous system associated with *Treponema Pallidum*, which can cause hearing loss and other neurological problems (21). In addition, it was shown that the electroacoustic response of children with the presence of congenital syphilis may be lower than that of children without RIHL (22), which may become one of the reasons for the detection of late hearing changes, usually discovered during auditory monitoring or when the child begins to present learning difficulties at school or even in speech, mainly due to changes in auditory skills not detected at the time of screening (23, 24).

Nevertheless, in relation to congenital syphilis, its presence can also lead to prematurity and the use of medication for the treatment of the mother and child when the infection is detected (1), which is precisely what was observed in the only case of referral for hearing diagnosis, which had the presence not only of congenital syphilis but also of other risk indicators, which may have increased the risk of hearing impairment since some of these are associated with failure in screening and hearing loss at birth (25) and, as observed, are often interconnected.

Another issue is the possibility of lower identification of other congenital infections, such as toxoplasmosis and congenital cytomegalovirus, that, in the data of this study, appeared, respectively, in the 14th and 18th positions among RIHL's percentage considering the entire sample for the 3 years observed. In 2021, congenital toxoplasmosis was incorporated into Brazil's Newborn Screening Program's Guthrie Test. This addition was prompted by its notable occurrence and its significant implications for child development (26). However, this new public policy does not reflect the data presented here but may, in the future, represent possible changes in these findings. Similar to congenital toxoplasmosis, congenital cytomegalovirus (CMV) may be underreported in the findings of this study because symptomatic and severe cases are identified and reported.

This context highlights the importance of monitoring these neonates after hearing screening and of public policies and guidelines to reduce the time for identification of hearing alterations and, consequently, the time for an intervention.

Internationally, the JCIH guidelines (4) direct NHSP, indicating the ideal moment of hearing screening, the period for diagnosis, and the conditions for hearing monitoring. In Brazil, the Guidelines for Neonatal Hearing Screening (11), the COMUSA technical note (8), and the National Plan for the Rights of Persons with Disabilities (27) present all the guidelines, directives, and laws about the hearing health of the Brazilian population, including children.

In addition to actions aimed at hearing health at international and national levels, the fight against syphilis, especially regarding the proper treatment during pregnancy, which is the time when maternal infection can occur in the fetus, is at risk of leading to numerous outcomes, such as abortion, prematurity, and hearing loss. The measures taken to tackle syphilis are the same as those of the Brazilian Unified Health System: prevention activities, early detection, and adequate treatment (1, 9).

Despite national efforts, the programs of neonatal hearing screening still cannot reach an adequate screening coverage rate and present great evasion in the following stages (28), which can compromise the early detection of hearing alterations in patients with risk indicators and also their follow-up (29). This aspect was observed by the coverage of 66.69% of those born in the public maternity ward having undergone neonatal hearing screening, when ideally, this coverage should be approximately 97% of newborns. This constituted a limitation of this study, as the 33.31% of newborns not tested in the hearing screening could have other outcomes. However, newborns with congenital syphilis spend an average of 10 days being treated in the maternity ward, which gives them a greater probability of being tested before hospital discharge. Thus, it is possible that a smaller number of newborns with congenital syphilis were not tested before hospital discharge. In addition, this structure that allows treatment when the infant has a title that indicates congenital syphilis means that all infants are treated, and the treatment is not subject to the return of the family to the service or administration of medication at home since hospital discharge is conditioned to treatment. Unfortunately, the maternity hospital does not have a computerized database that allows us to analyze newborns not tested in the hearing screening. The database used for this study was from the Maternity Hearing Screening Sector, therefore, including all those who underwent hearing screening.

Another possible cause of the limited coverage of the NHS was professional work arrangements, which do not include professionals every weekend. These children who were discharged from maternity hospitals without the NHS had scheduled ambulatory appointments. Considering common vulnerabilities in this population (limited awareness of the importance of this evaluation and a place of residence far from the maternity hospital), we can find another possible cause of this coverage. Despite the limitations of the study's sample on coverage of the NHS and risk factors, a statistical analysis was performed to reduce impacts and highlight the main points that require careful attention from management on children's health, as discussed below.

Furthermore, the lack of data control and the need for unified computerization of data hinder the follow-up of these children in the stages of monitoring and diagnosis of services (30).

Public policy efforts to prevent and combat syphilis are also necessary to continually reduce the incidence rate of syphilis in pregnant women and cases of congenital syphilis. These efforts also play a crucial role in promoting accurate detection and adequate treatment for infected mothers and children. This approach seeks to minimize the impact of congenital syphilis, particularly on hearing and child development outcomes.

A study conducted by Brito et al. (31) revealed that both Brazil and Portugal have established adequate protocols for the identification and treatment of syphilis in pregnant women and of cases of congenital syphilis. However, the incidence is higher in Brazil, influenced significantly by socioeconomic, cultural, and social factors, as well as the vastness of its territory in comparison to Portugal. This situation is aggravated by issues within Brazil's prenatal care system, including challenges in monitoring pregnant women and the integration of information between epidemiological surveillance and primary health care. The Brazilian health information systems related to syphilis contribute to the fragmentation of health data and information, delays in diagnosis, management of incomplete cases, and loss of data due to inconsistencies and inadequate reporting (31).

Moreover, it is crucial to emphasize the need for reinforcing the healthcare networks within the health system that oversee the follow-up of children with congenital syphilis during outpatient visits, either in primary and specialized care settings. This approach aims to prevent families from slipping through the cracks and to ensure that early childhood hearing loss is promptly detected without any hindrance (32).

Finally, it was observed that there are two limiting conditions in the study: neonates who did not undergo outpatient testing and/or retesting due to non-attendance or opting for testing at a different facility and neonates who need to undergo hearing diagnosis from an alternative hearing healthcare service. These groups were excluded from the study, resulting in the absence of corresponding results for this aspect.

It is suggested that further research be done on the presence of congenital syphilis after neonatal hearing screening to verify whether the presence of this risk indicator is, in fact, a risk for the development of future hearing loss. There should also be studies that can observe the potential of two or more indicators for hearing loss in childhood.

If there is a low risk for failure in neonatal hearing screening in newborns with congenital syphilis and, in future follow-up studies, a low risk of the presence of hearing impairment in infants with congenital syphilis treated at birth can lead to changes in management and guidelines in the care network for these infants compared to others with a higher prevalence of failure and of triggering hearing loss that, currently, are not included in the public policies in Brazil and elsewhere in the world.

Public health practices have evolved over time, and in Brazil, a notable change has occurred where newborns identified and diagnosed with congenital syphilis at birth have access to treatment before being discharged from the hospital; this positive development contrasts with the treatment observed in previous decades and even in the present situation in certain regions around the world. Therefore, this study can help alert the scientific society and managers in the health area to the importance of identifying the epidemiological data of each reality in view of the public policies adopted to adapt identification and follow-up behaviors in health.

This study shows a greater risk of screening failure in the presence of two or more risk indicators (co-occurrence), hence the importance of monitoring these newborns more closely after neonatal hearing screening. The strengths of the study also include a large sample and a variety of RIHL. However, the incidence percentage of passing and failing on the NHS shows similar risk factors. This result points to the need to study not only isolated risk factors but also their co-occurrence, considering local characteristics such as socioeconomic ones.

Among the RIHL observed in the sample, prematurity was more frequent in the newborns who passed the screening (55.26%) than in those who failed the test (45.67%). Since the maternity hospital in the study is a high-risk hospital and a reference in the state, the volume of patients with risk conditions increases, and it is more common to have those born prematurely. Owing to this, we can observe a homogeneous distribution of this risk indicator in the sample.

A lack of standardized protocols is evident in neonatal hearing screening practices (33, 34). Despite this, there is a prevailing tendency toward recommending protocols involving the application of AABR due to its demonstrated higher specificity and reduced false positives in detecting hearing loss. This contrasts with protocols employing solely TEOAE in the first stage, followed by AABR in the second stage (35). Similar results were observed, indicating that the inclusion of AABR can significantly reduce referral rates without increasing diagnostic error rates. However, this improvement comes at a higher cost compared to using TEOAE alone, but it also offers greater accuracy (36).

In addition, the maternity hospital in this study uses TEOAE for hearing screening across all sectors. The Joint Committee on Infant Hearing (4) advocates the use of ABR in neonatal ICUs while recommending the use of TEOAE for other sectors. Our study's sample aligns with this approach, given that most infants with congenital syphilis were not in NICUs. The use of TEOAE did not influence the hearing screening outcomes, which is consistent with findings from a study that used AABR (7). One factor that must be considered is that AABR requires qualified professionals and incurs greater costs. Thus, the recommendation emphasizes TEOAE for newborns without known risks and reserves AABR for those at risk (37).

## Conclusions

Congenital syphilis was the ninth most frequent risk indicator for hearing loss and, in isolation, does not present a risk for failure in neonatal hearing screening since no neonate was referred for audiological diagnosis. Notably, congenital syphilis can cause late hearing loss during child development; thus, audiological monitoring of these neonates and studies that address this issue are recommended.

## Data availability statement

The raw data supporting the conclusions of this article will be made available by the authors, without undue reservation.

## Ethics statement

The studies involving humans were approved by University Hospital Onofre Lopes of the Federal University of Rio Grande do Norte. The studies were conducted in accordance with the local legislation and institutional requirements. Written informed consent for participation was not required from the participants or the participants' legal guardians/next of kin because we collect the information in the Institution's database, without access to personal information.

## Author contributions

TO, MD, AN-A, and AS performed the collection, analysis, tabulation of data and writing, and revision of the manuscript. GO, GS, and RV performed analysis data and scientific writing. SB conceived and guided the study and was responsible for analyzing data, writing, and revising the manuscript. All authors contributed to the article and approved the submitted version.
